# Breast conservation therapy decreased heart-specific mortality in breast cancer patients compared with mastectomy

**DOI:** 10.1186/s12893-023-02132-1

**Published:** 2023-08-12

**Authors:** Zhi Li, Ruipeng Zhao

**Affiliations:** Department of Thyroid and Breast Surgery, The Affiliated Huaian No. 1 People’s Hospital of Nanjing Medical University, Huaian, Jiangsu China

**Keywords:** Breast cancer, Breast conservation therapy, Mastectomy, Cardiotoxicity, SEER

## Abstract

**Aim:**

To investigate the impacts of breast conservation therapy (BCT) and mastectomy on heart-specific mortality in breast cancer patients.

**Methods:**

Patients with primary breast cancer registered in the Surveillance, Epidemiology, and End Results (SEER) database between Jan 1998 and Dec 2015 were included. Patients were divided into either breast conservation therapy or mastectomy group. To compare mortality caused by heart diseases in breast cancer patients with BCT or mastectomy, univariate and multivariate regression after propensity score matching (PSM) were performed. Kaplan-Meier analysis was also used to evaluate heart-specific survival between two groups.

**Results:**

132,616 patients with breast cancer were enrolled in this study. After PSM, four risk factors including age, race, marital status and types of surgery were identified significantly associated with death from heart diseases. Heart-specific survival analysis further showed that overall, BCT poses a lower risk to heart-specific mortality compared with mastectomy.

**Conclusion:**

Compared with mastectomy, BCT significantly decreased heart-specific mortality in breast cancer patients.

## Introduction

Breast cancer is currently the most common women’s cancer, with an estimated 2 million new diagnosis in 2018 globally, and the fifth most common cause of mortality from cancer [1]. Progress made in treatment of breast cancer has largely improved the prognosis in breast cancer. A 41% reduction of mortality rate compared with that in 1990s was reported for 2021 [2]. However, despite better survival, complications from these treatments are also increasing. Studies have shown that patients with breast cancer could develop cardiovascular diseases (CVD) during treatment because of the cardiotoxicity from chemo-or radio-therapy and targeted therapy (e.g., anti-HER2 therapeutics) [3–6], and CVD has become an important factor in all-cause mortality of breast cancer patients [7]. Similarly, we analyzed the data from Surveillance, Epidemiology, and End Results Program (SEER) database and found that heart-specific is the second most common cause of death in patients with breast cancer. Furthermore, it was found that in elderly breast cancer patients, especially patients with preexisting heart diseases, heart-specific disease has become the leading cause of death, while breast cancer comes the second [8]. There is also evidence suggesting that other than age, race and cancer stage are also risk factors associated with CVD mortality [9].

Currently, surgery including mastectomy and breast conserving therapy (BCT), with chemoradiotherapy, endocrine therapy and molecular targeted therapy, is the standard of care in treating breast cancer [10]. Due to the development of radiotherapy, BCT is now more widely performed [11, 12]. No significant difference of prognosis, including overall survival and disease-free interval, was observed in previous studies, comparing BCT combining radiotherapy to mastectomy [13–16].Recent results also showed that BCT might be more beneficial in terms of overall survival [17, 18]. Therefore, it was suggested BCT might be preferred over mastectomy. However, whether BCT and mastectomy would impact on heart-specific mortality in breast cancer patients is still unclear. Here, using SEER database, we assess the death resulted from heart disease in breast cancer patients who had underwent either BCT or mastectomy, to further demonstrate if the BCT would outweigh mastectomy regarding heart-specific survival.

## Methods

### Patients

**Fig. 1:**
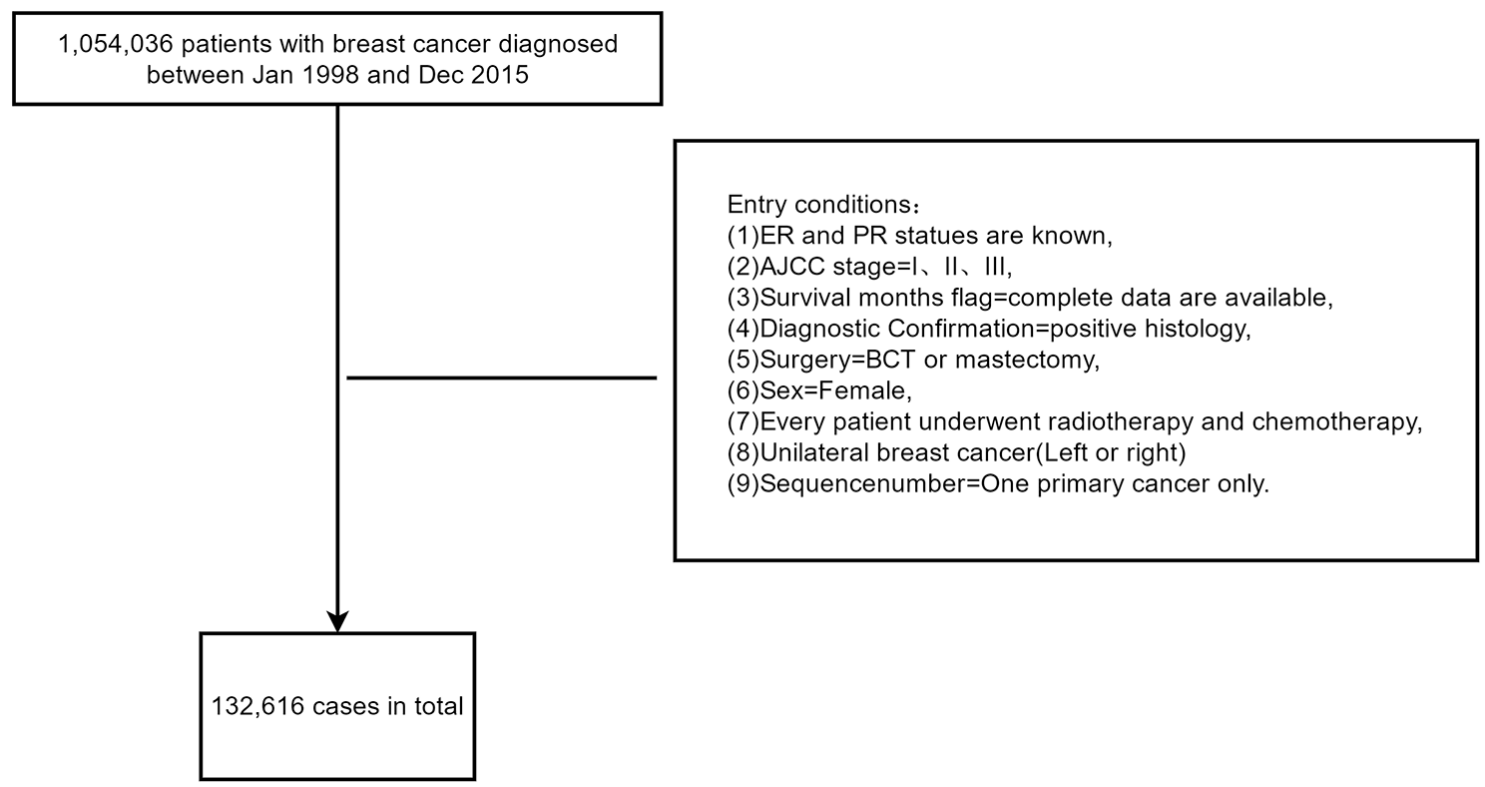
The flow chart for entry conditions

The data were derived from the SEER database between Jan 1998 and Dec 2015 using SEER*Stat v8.3.5 software. Only patients with primary breast cancer were selected. The criteria are as followed: age at diagnose, sex, race, marital status, type of surgery, American Joint Committee on Cancer (AJCC) stage, estrogen receptor (ER) and progestogen receptor(PR) status, laterality, cause of mortality, survival time. The flow chart for entry conditions were shown in Fig. 1.

### Statistical analysis

The primary endpoint is heart-specific survival (HSS), defined as the time between confirmed heart diseases diagnosis to death from them or last follow up. The heart diseases were listed in Table 1. Kaplan-Meier analysis was performed to estimate HSS. Cox proportional-hazards models were used to estimate hazard ratio and 95% confidence intervals within the same subgroup. Competing risk model was developed in R v3.6.2 via using package “cmprsk”, the primary endpoint of competing risk model is survival between the time at diagnosis of any diseases to death. The other analysis such as propensity score matching (PSM) were performed by SPSS 25.0. A p value less than 0.01 was considered significant.

**Table 1:** Heart-specific causes of death encoded with International Classification of Diseases 10 (ICD-10) codes in SEER database

Causes of Death	SEER Code	Medical Term	ICD-10 Code
Diseases of Heart	50,060	Ischaemic heart diseases	I20-I25
		Hypertensive heart disease	I11, I13
		Other forms of heart disease (e.g., heart failure)	I30-I51
		Pulmonary heart disease and diseases of pulmonary circulation	I26-I28
		Rheumatic heart disease	I00-I02, I05-I09

## Results

### Study population

A total of 132,616 patients were included in this study. The demographic and clinical characteristics were described in Table 2. Overall, 47,308 (35.7%) patients had mastectomy and 85,308 (64.3%) had BCT. The average follow-up time was 88.4 months. The mean age at diagnosis of breast cancer was 53.26 years old. Majority of the patients were white population (77.2%) and the black population only accounted for 13.2%, with the rest (including Latinos and Asians) being 9.3%. For AJCC stage, 89.2% of the patients were at either stage I or stage II when first diagnosed. More than half of patients were either ER or PR positive.

**Table 2:** Baseline of patients characteristics

characteristics	N = 132,616	Mastectomy N = 47,308(35.7%) N (%)	BCT N = 85,308(64.3%) N (%)	P value
Age at diagnosis (year)				
≤50	55,601(41.9%)	22,378(47.3%)	33,223(38.9%)	<0.0001
>50	77,015(58.1%)	24,930(52.7%)	52,085(61.6%)	
Race				
White	102,334(77.2%)	35,967(76%)	66,367(77.8%)	<0.0001
Black	17,554(13.2%)	6440(13.6%)	11,114(13%)	
Others^a^	12,296(9.3%)	4768(10.1%)	7528(8.8%)	
Unknown	432(0.3%)	133(0.3%)	299(0.4%)	
AJCC Stage				
I	32,445(24.5%)	1459(3.1%)	30,986(36.3%)	<0.0001
II	63,391(47.8%)	18,278(38.6%)	45,113(52.9%)	
III	36,780(27.7%)	27,571(58.3%)	9209(10.8%)	
ER status				
Negative	40,443(30.5%)	13,641(28.8%)	26,802(31.4%)	<0.0001
Positive	92,173(69.5%)	33,667(71.2%)	58,506(68.6%)	
PR status				
Negative	54,425(41%)	18,956(40.1%)	35,469(41.6%)	<0.0001
Positive	78,191(59%)	28,352(59.9%)	49,839(58.4%)	
Marital status				
Married	83,098(62.7%)	29,055(61.4%)	54,043(63.4%)	<0.0001
Widowed	8460(6.4%)	3213(6.8%)	5247(6.2%)	
Others^b^	36,779(27.7%)	13,507(28.6%)	23,272(27.3%)	
Unknown	4279(3.2%)	1533(3.2%)	2746(3.2%)	
Laterality				
Left	67,337(50.8%)	23,816(50.3%)	43,521(51%)	0.019
Right	65,279(49.2%)	23,492(49.7%)	41,787(49%)	

a, other includes Latinos, Alaskan native, Asian, American Indian and Pacific Islander. b, others includes divorced, single and unmarried

### Competing risk mortality

**Fig. 2:**
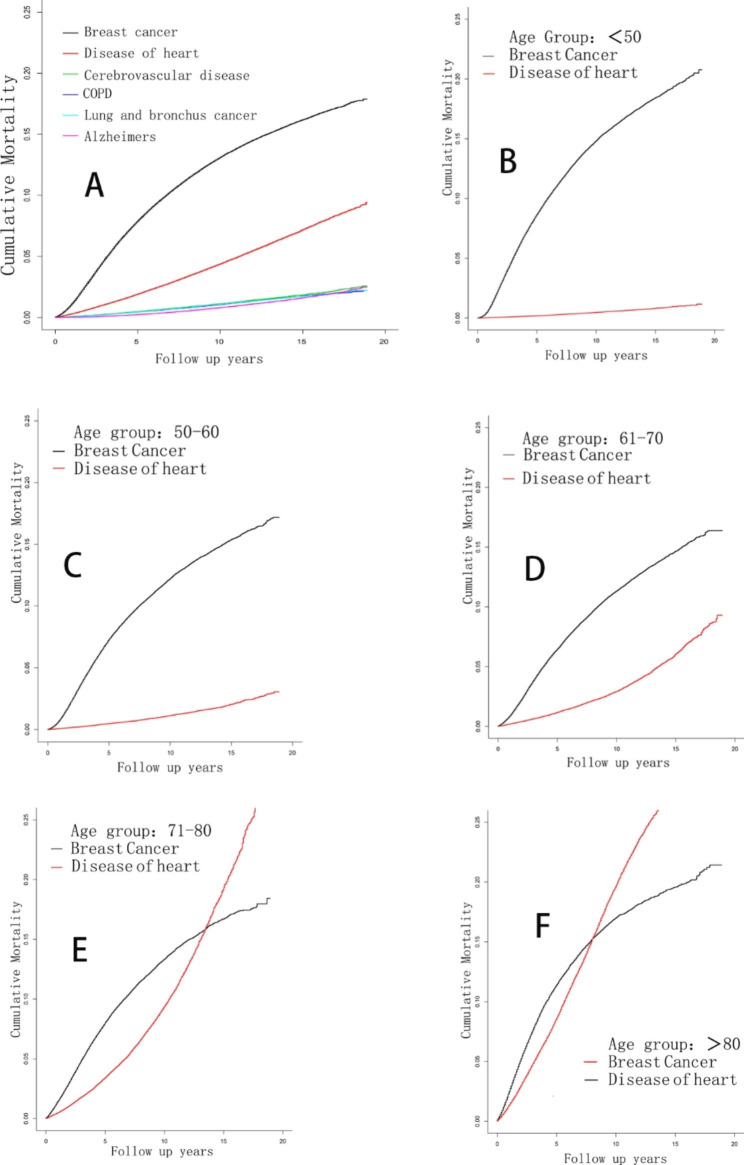
A. Overall competing risk analysis. B, C, D, E and F. Age-subgroup analysis of risk of heart-related death

As shown in Fig. 2, heart-specific mortality was only second to breast cancer in terms of cause of mortality overall. Age-subgroup analysis further demonstrated that the risk of death from diseases of heart increased with age. In subgroup aged between 71 and 80, the mortality rate resulted from diseases of heart was higher than that caused by breast cancer after 13-year follow up. In subgroup aged 80 and over, after 8-year follow up, the heart-specific mortality rate was the leading cause of death, while breast cancer mortality ranked second.

### Kaplan-Meier HSS in breast cancer patients

**Fig. 3:**
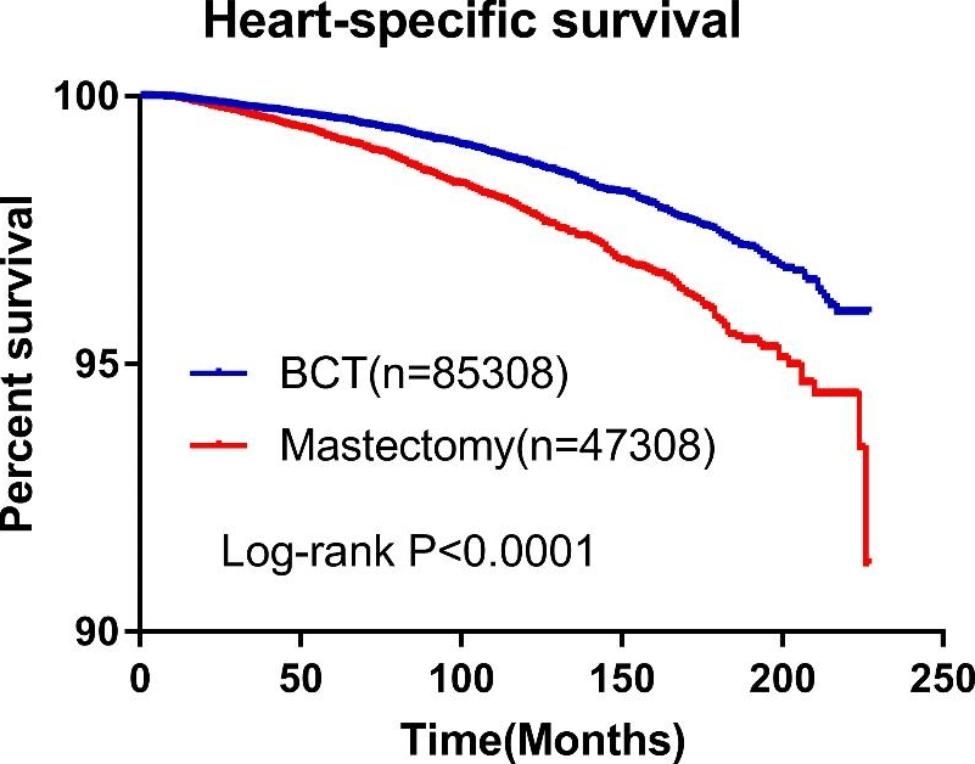
Kaplan-Meier curve of HSS in BCT group and mastectomy group. It indicated that BCT had a lower risk of developing heart-specific death compared with mastectomy (P < 0.0001)

It showed that compared with HSS in mastectomy, HSS in BCT was significantly higher (P < 0.0001) (Fig. 3).

### Risk factors for HSS identified by univariate and multivariate Cox analysis

As shown in Table 3,univariable analysis found that age, race, AJCC staging, ER and PR status, marital status, laterality and types of surgery were risk factors for HSS. Multivariable analysis was performed to further identify the variables mentioned above. Compared with patients aged no more than 50 at diagnosis, patients older than 50 were at a higher risk of heart-specific death (HR = 4.423, P < 0.0001). We also found that black populations had the highest risk of heart-specific mortality (HR = 1.647, P < 0.0001). Unsurprisingly, late stage posed a risk to death from heart disease (stage II: H = 1.348, P < 0.0001; stage III: HR = 1.935, P < 0.0001). No difference was observed between ER or PR status in heart-specific mortality. In terms of marital status, married patients were found to be less likely to die of heart-disease. Compared with BCT, mastectomy was significantly associated with higher risk of death from heart diseases(HR = 1.364, P < 0.0001).

**Table 3:** Cox proportional-hazards models of HSS

Variables	Univariable analysis			multivariable analysis		
	HR	95%CI	P value	HR	95%CI	P value
Age at diagnosis (year)						
≤50	Ref			Ref		
>50	4.972	4.305–5.742	<0.0001	4.423	3.818–5.123	<0.0001
Race						
White	Ref			Ref		
Black	1.74	1.525–1.987	<0.0001	1.647	1.438–1.886	<0.0001
Others^a^	0.68	0.548–0.842	<0.0001	0.771	0.622–0.956	0.018
Unknown	0	0-1.45E + 33	0.836	0	0-3.02E + 37	0.852
AJCC Stage						
I	Ref			Ref		
II	1.444	1.254–1.663	<0.0001	1.348	1.166–1.557	<0.0001
III	2.467	2.132–2.855	<0.0001	1.935	1.63–2.297	<0.0001
ER status						
Negative	Ref			Ref		
Positive	0.806	0.724–0.898	<0.0001	0.892	0.766–1.038	0.138
PR status						
Negative	Ref			Ref		
Positive	0.742	0.67–0.822	<0.0001	0.874	0.758–1.009	0.066
Marital status						
Married	Ref			Ref		
Widowed	5.408	4.737–6.175	<0.0001	3.377	2.95–3.866	<0.0001
Others^b^	1.525	1.352–1.721	<0.0001	1.45	1.283–1.64	<0.0001
Unknown	1.662	1.237–2.234	0.001	1.533	1.14–2.061	0.005
Laterality						
Left	Ref			Ref		
Right	0.976	0.881–1.08	0.634	0.977	0.883–1.082	0.659
Type of surgery						
BCT	Ref			Ref		
Mastectomy	1.703	1.535–1.888	<0.0001	1.364	1.204–1.545	<0.0001

a, other includes Latinos, Alaskan native, Asian, American Indian and Pacific Islander. b, others includes divorced, single and unmarried

### K-M survival analysis and multivariable analysis after propensity score matching (PSM)

**Fig. 4:**
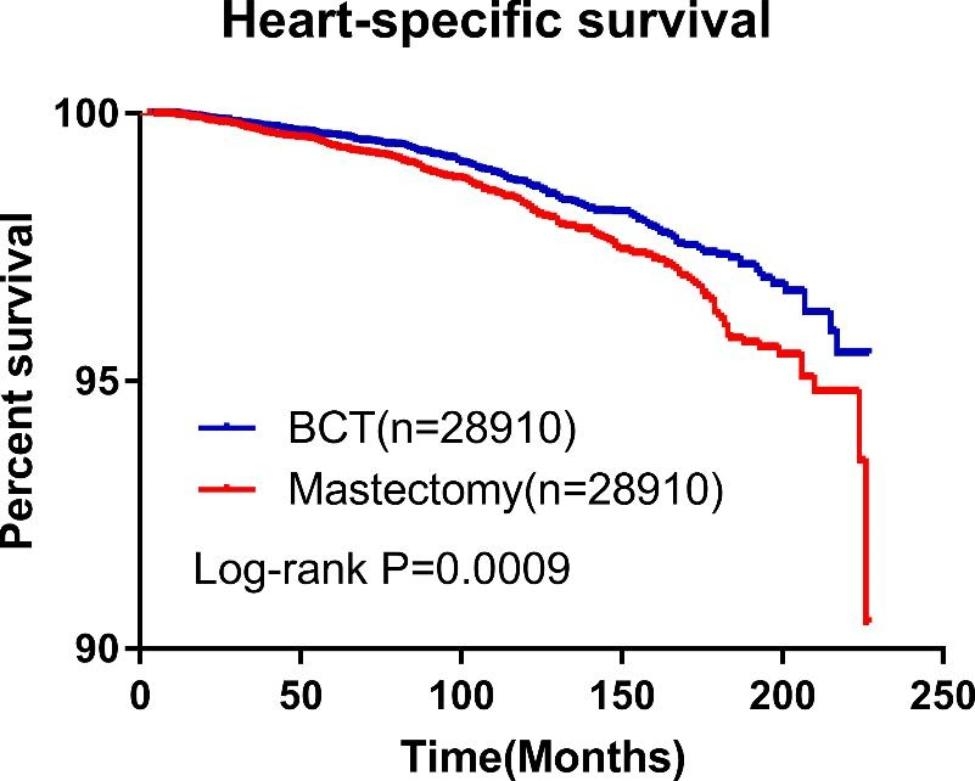
Kaplan-Meier curve of HSS in BCT group and mastectomy group after PSM. It indicated that BCT had a significantly lower risk of developing heart-specific death compared with mastectomy (P = 0.0009)

To adjust the imbalance between groups, PSM was performed to age, race, AJCC staging, ER and PR status, marital status and laterality (Table 4). Survival analysis after PSM showed HSS was significantly higher in BCT group compared with mastectomy (P = 0.0009) (Fig. 4). The same result was also observed after adjusting for the other variables (HR = 1.311, P < 0.001). In addition, age over 50 (HR = 4.001, p < 0.0001) and black population (HR = 1.855, p < 0.0001) were also found as risk factors for heart-specific death (Table 5), while being married was protective.

**Table 4:** Patient characteristics after PSM

Variables	N = 57,820	Mastectomy N = 28,910 N (%)	BCT N = 28,910 N (%)	P value
Age at diagnosis (year)				1
≤50	27,442(47.5%)	13,721(47.5%)	13,721(47.5%)	
>50	30,378(52.5%)	15,189(52.5%)	15,189(52.5%)	
Race				
White	43,713(75.6%)	21,856(75.6%)	21,857(75.6%)	0.996
Black	8226(14.2%)	4115(14.2%)	4111(14.2%)	
Others^a^	5722(9.9%)	2858(9.9%)	2864(9.9%)	
Unknown	159(0.3%)	81(0.3%)	78(0.3%)	
AJCC Stage				
I	2916(5%)	1458(5%)	1458(5%)	1
II	36,518(63.2%)	18,259(63.2%)	18,259(63.2%)	
III	18,386(31.8%)	9193(31.8%)	9193(31.8%)	
ER status				
Negative	16,818(29.1%)	8409(29.1%)	8409(29.1%)	1
Positive	41,002(70.9%)	20,501(70.9%)	20,501(70.9%)	
PR status				
Negative	22,926(39.7%)	11,463(39.7%)	11,463(39.7%)	1
Positive	34,894(60.3%)	17,447(60.3%)	17,447(60.3%)	
Marital status				
Married	36,086(62.4%)	18,043(62.4%)	18,043(62.4%)	0.998
Widowed	3535(6.1%)	1768(6.1%)	1767(6.1%)	
Others^b^	16,337(28.3%)	8172(28.3%)	8165(28.3%)	
Unknown	1862(3.2%)	927(3.2%)	935(3.2%)	
Laterality				
Left	29,114(50.4%)	14,554(50.4%)	14,560(50.4%)	0.960
Right	28,706(49.6%)	14,356(49.6%)	14,350(49.6%)	

a, other includes Latinos, Alaskan native, Asian, American Indian and Pacific Islander. b, others includes divorced, single and unmarried

**Table 5:** Cox proportional-hazards models of HSS after PSM

Variables	Univariable analysis			Multivariable analysis		
	HR	95%CI	P value	HR	95%CI	P value
Age at diagnosis (year)						
≤50	Ref			Ref		
>50	4.786	3.921–5.843	<0.0001	4.001	3.26–4.91	<0.0001
Race						
White	Ref			Ref		
Black	1.968	1.632–2.372	<0.0001	1.855	1.531–2.248	<0.0001
Others^a^	0.709	0.519–0.969	0.031	0.86	0.629–1.176	0.345
Unknown	0	0-1.32E + 57	0.9	0	0-5.09E + 41	0.876
AJCC Stage						
I	Ref			Ref		
II	1.637	1.055–2.542	0.028	1.548	0.997–2.405	0.052
III	2.657	1.704–4.144	<0.0001	2.125	1.361–3.318	0.001
ER status						
Negative	Ref			Ref		
Positive	0.753	0.64–0.885	0.001	0.914	0.73–1.144	0.431
PR status						
Negative	Ref			Ref		
Positive	0.663	0.568–0.772	<0.0001	0.759	0.614–0.938	0.31
Marital status						
Married	Ref			Ref		
Widowed	5.442	4.438–6.672	<0.0001	3.326	2.702–4.095	<0.0001
Others^b^	1.668	1.395–1.994	<0.0001	1.505	1.254–1.806	<0.0001
Unknown	1.611	1.024–2.534	0.039	1.467	0.932–2.309	0.098
Laterality						
Left	Ref			Ref		
Right	0.941	0.807–1.096	0.433	0.941	0.817–1.099	0.441
Type of surgery						
BCT	Ref			Ref		
Mastectomy	1.367	1.173–1.594	<0.0001	1.311	1.125–1.529	0.001

a, other includes Latinos, Alaskan native, Asian, American Indian and Pacific Islander. b, others includes divorced, single and unmarried

## Discussion

To provide a high-quality and patient-centered care, more attention should also be paid to health issues beyond breast cancer during treatment. This study is a large retrospective cohort study. In this study, we identified that heart-specific mortality is high in breast cancer patients, only second to breast cancer itself.

It was reported that the confirmation and treatment of breast cancer would induce posttraumatic stress disorder (PTSD), which has been known highly associated with death from coronary heart diseases and cardiovascular events [19–24]. That is, breast cancer could have a negative impact on cardiovascular health.

In line with previous studies[25–27], older breast cancer patients were more likely to die of heart diseases rather than breast cancer, and such risk increased with age. Previously, Safford et al. showed that compared with others (e.g., white, Asian and Hispanic women), black women experienced a higher risk of death from heart diseases [28]. The mortality rate was also higher in black females after taking treatment into account [29]. In this study, we also found the black women had a higher heart-specific mortality than the others. There might be several reasons for that, such as lower social-economic status. In terms of marital status, married women had the lowest risk. As reported by Dupre et al. [30], among patients with acute myocardial infarction, married patients had the lowest risk of death compared with other marital statuses. Schaal S et al. [31]suggested that widowhood could cause depression combined with PTSD which has been deemed a risk factor for coronary heart disease [23]. Besides, McGarry Kathleen et al. found that the number of widowhood below the poverty threshold was three times that of matrimony, which retained them from seeking better medical treatment [32, 34]. Therefore, lack of both psychological and financial support from partners also could pose a higher risk to heart-specific death.

In this study, patients with BCT were found to have a lower risk of death from heart diseases than patients with mastectomy. It might because BCT is less traumatic and takes a shorter recovery time postoperatively. A study by Šimunović, M. et al. suggested that patients who underwent BCT had lower trauma stress response scores [33]. It might also easier for BCT patients to return to normal life as less changes would be made to breast. A study including 3233 women with breast cancer by Flanagan, M. R. et al. showed that compared with patients with mastectomy, BCT patients had a higher satisfaction rate and higher quality of life [34, 13].

Regarding the surgical aspect, studies are not conclusive as to the improved survival rates in metastatic breast cancer patients undergoing resection [35].In this study, the risk of death attributable to heart diseases was higher in late stage breast cancer. That might be due to more chemoradiotherapy and higher doses they had received, leading to more iatrogenic cardiotoxicity, and more PTSD as well.

Interestingly, while it has been recognized that estrogen has a protective effect on cardiovascular system and anti-estrogen therapy may thus negatively impact on that [36, 37], It was also reported correlations between endocrine pathologies as negative prognostic factors both for breast cancer progression and survival [38],our study showed no significant difference in the risk of death from heart diseases between ER or PR positive and negative subgroups. More research may be required to clarify the underlying mechanisms.

This study also has some limitations. First, this study is a retrospective study so the data quality and reliability may be limited. Second, it is confirmed that chemotherapy and targeted therapy can cause either early or delayed cardiotoxicity, especially anthracyclines and Trastuzumab. However, due to lack of data about chemotherapy regimens and use of Trastuzumab in SEER database, we were not able to further explore the effect of chemotherapeutics and Trastuzumab on heart-specific mortality [39–44]. What’s more, while comorbidities such as diabetes and hypertension that could also contribute to heart diseases, our study didn’t include these preexisting conditions because of insufficient data from SEER.

## Conclusion

To conclude, our study showed that compared with mastectomy, BCT posed a significantly lower risk of heart-specific mortality. Age, race, stage and marital status were also important risk factors of death caused by heart diseases .

## Data Availability

The database generated during the current study are available in SEER(Surveillance, Epidemiology, and End Results Program) database.(https://seer.cancer.gov/).
